# Integrated dynamic wet spinning of core-sheath hydrogel fibers for optical-to-brain/tissue communications

**DOI:** 10.1093/nsr/nwaa209

**Published:** 2020-08-31

**Authors:** Guoyin Chen, Gang Wang, Xinrong Tan, Kai Hou, Qingshuo Meng, Peng Zhao, Shun Wang, Jiayi Zhang, Zhan Zhou, Tao Chen, Yanhua Cheng, Benjamin S Hsiao, Elsa Reichmanis, Meifang Zhu

**Affiliations:** State Key Laboratory for Modification of Chemical Fibers and Polymer Materials, College of Materials Science and Engineering, Donghua University, Shanghai 201620, China; State Key Laboratory for Modification of Chemical Fibers and Polymer Materials, College of Materials Science and Engineering, Donghua University, Shanghai 201620, China; State Key Laboratory of Medical Neurobiology, MOE Frontiers Center for Brain Science, Institute of Brain Science, Department of Ophthalmology, Zhongshan Hospital, Fudan University, Shanghai 200032, China; State Key Laboratory for Modification of Chemical Fibers and Polymer Materials, College of Materials Science and Engineering, Donghua University, Shanghai 201620, China; State Key Laboratory of Medical Neurobiology, MOE Frontiers Center for Brain Science, Institute of Brain Science, Department of Ophthalmology, Zhongshan Hospital, Fudan University, Shanghai 200032, China; State Key Laboratory of Medical Neurobiology, MOE Frontiers Center for Brain Science, Institute of Brain Science, Department of Ophthalmology, Zhongshan Hospital, Fudan University, Shanghai 200032, China; State Key Laboratory for Modification of Chemical Fibers and Polymer Materials, College of Materials Science and Engineering, Donghua University, Shanghai 201620, China; State Key Laboratory of Medical Neurobiology, MOE Frontiers Center for Brain Science, Institute of Brain Science, Department of Ophthalmology, Zhongshan Hospital, Fudan University, Shanghai 200032, China; State Key Laboratory for Modification of Chemical Fibers and Polymer Materials, College of Materials Science and Engineering, Donghua University, Shanghai 201620, China; State Key Laboratory for Modification of Chemical Fibers and Polymer Materials, College of Materials Science and Engineering, Donghua University, Shanghai 201620, China; State Key Laboratory for Modification of Chemical Fibers and Polymer Materials, College of Materials Science and Engineering, Donghua University, Shanghai 201620, China; State Key Laboratory for Modification of Chemical Fibers and Polymer Materials, College of Materials Science and Engineering, Donghua University, Shanghai 201620, China; Department of Chemistry, Stony Brook University, Stony Brook, NY 11794, USA; School of Chemical and Biomolecular Engineering, School of Chemistry and Biochemistry, School of Materials Science and Engineering, Georgia Institute of Technology, Atlanta, GA 30332, USA; State Key Laboratory for Modification of Chemical Fibers and Polymer Materials, College of Materials Science and Engineering, Donghua University, Shanghai 201620, China

**Keywords:** optical waveguide, hydrogel fiber, deep-tissue photothermal therapy, optogenetic stimulation

## Abstract

Hydrogel optical light-guides have received substantial interest for applications such as deep-tissue biosensors, optogenetic stimulation and photomedicine due to their biocompatibility, (micro)structure control and tissue-like Young's modulus. However, despite recent developments, large-scale fabrication with a continuous synthetic methodology, which could produce core-sheath hydrogel fibers with the desired optical and mechanical properties suitable for deep-tissue applications, has yet to be achieved. In this study, we report a versatile concept of integrated light-triggered dynamic wet spinning capable of continuously producing core-sheath hydrogel optical fibers with tunable fiber diameters, and mechanical and optical propagation properties. Furthermore, this concept also exhibited versatility for various kinds of core-sheath functional fibers. The wet spinning synthetic procedure and fabrication process were optimized with the rational design of the core/sheath material interface compatibility [core = poly(ethylene glycol diacrylate-*co*-acrylamide); sheath = Ca-alginate], optical transparency, refractive index and spinning solution viscosity. The resulting hydrogel optical fibers exhibited desirable low optical attenuation (0.18 ± 0.01 dB cm^−1^ with 650 nm laser light), excellent biocompatibility and tissue-like Young's modulus (<2.60 MPa). The optical waveguide hydrogel fibers were successfully employed for deep-tissue cancer therapy and brain optogenetic stimulation, confirming that they could serve as an efficient versatile tool for diverse deep-tissue therapy and brain optogenetic applications.

## Teaser

A concept of integrated light-triggered dynamic wet spinning is demonstrated for continuous synthesis of core-sheath hydrogel optical fibers, which could serve as a versatile tool for deep-tissue biomedical applications.

## INTRODUCTION

Hydrogel fiber-based optical light-guides have received considerable attention for applications in biosensors, optogenetic stimulation and deep-tissue photomedicine due to their excellent biocompatibility and tissue-like Young's modulus [1–5]. Some notable examples of polymers in this family include the Ca-alginate/acrylamide (AAm) hydrogel fibers used for brain-optical communications [6] and glucose-sensitive hydrogel fibers used as implantable glucose sensors [7]. Compared to traditional optical fibers (e.g. glass and polymer-based optical fibers), core-sheath hydrogel optical fibers have a soft, wet nature and can offer certain distinct advantages such as superior mechanical properties and host-like biocompatibility, especially for implantable biomedical applications [8]. To date, however, most demonstrated fabrication procedures have involved small-scale template assembly [3,6,7,9,10], which indicates a severe limitation of this fiber class for applications requiring large-scale synthesis/manufacturing.

The presence of crystalline phases in an optical waveguide is well known for being able to cause opacity due to light scattering at the interface between crystalline and amorphous domains with different refractive indexes. Thus, realizing amorphous crosslinked polymer structures from monomers or polymers is an effective way to produce transparent materials for hydrogel-based optical fiber manufacturing. Despite recent developments in hydrogel fiber fabrication strategies, such as electrospinning [11], 3D printing [12,13], extrusion [14], microfluidic technology (combined with hydrodynamic focusing) [15] and the template method [6,7], the continuous and large-scale integrable production of core-sheath hydrogel fibers with the desired optical and mechanical properties remains challenging, mainly due to the following: (i) the simultaneous formation of the hydrogel network during the fiber fabrication process (poor processability of the stable crosslinked network after hydrogel gelation) [15–17]; (ii) different rheological behavior between core and sheath materials that often induces a non-equilibrium state during fiber spinning; (iii) the rational and generalizable selection of core and sheath materials required to obtain total reflection at the core/sheath interface.

Based on the above considerations as well as the existing mature fabrication, technology and controllability, wet spinning has been widely utilized for the production of polymer fibers on a large scale [18–21]. Prominent examples include commercial polymer fibers (e.g. polyvinyl alcohol and polyacrylonitrile), organic/inorganic hybrid fibers and hydrogel fiber from soluble polymers (e.g. Na-alginate and cellulose). Compared with the widely reported hydrogel fibers fabricated by wet spinning of polymer solutions, hydrogel fibers produced by dynamic-crosslinking-spinning [16,17,22] and microfluidic-spinning [15] from the poly(ethylene glycol) diacrylate (PEGDA) monomer have offered efficient pathways for fiber structural design, functionalization and scalable fabrication. An important common feature of the two methodologies is the use of a stable monomer solution trickle that can produce hydrogel fibers by UV induced polymerization. However, both the above methodologies remain confined to the fabrication of a single phase hydrogel fiber, and thus, their applicability to multiple phase fiber systems, especially the widely required core-sheath fibers, is a pressing question. Considering the core-sheath hydrogel fiber fabrication process, it appears quite challenging to formulate a methodology capable of assembling core and sheath structures into hydrogel fibers with crosslinked networks from monomers.

In this study, we report a concept of integrated light-triggered dynamic wet spinning (ILDWS) capable of continuously synthesizing core-sheath hydrogel optical fibers with a finely tuned fiber diameter, and mechanical and optical propagation properties. Furthermore, this concept is also suitable for many kinds of monomers to fabricate different core-sheath hydrogel fibers, such as p(PEGDA-*co*-AAm)/Ca-alginate-based semi-interpenetrating hydrogel fibers and p(NIPAm-*co*-DMAAm)-based hydrogel fibers. The wet spinning process was optimized with the rational design of the core/sheath material interface compatibility [core = p(PEGDA-*co*-AAm), sheath = Ca-alginate], optical transparency, refractive index and spinning solution viscosity. The fabricated hydrogel optical fibers exhibit desirable low optical attenuation (0.18 ± 0.01 dB cm^−1^ with 650 nm laser illumination, one of the lowest optical attenuations reported for hydrogel fiber materials), excellent biocompatibility and tissue-like Young's modulus (<2.60 MPa). The resulting fibers have been utilized for deep-tissue cancer therapy and brain optogenetic stimulation, and the optical waveguide hydrogel fiber (OWHF) in this work shows a better long-term mechanical compatibility and biocompatibility with brain tissue than the silica fiber, demonstrating that our hydrogel optical fiber could serve as an efficient and versatile tool for deep-tissue biomedical applications.

## RESULTS AND DISCUSSION

### Continuous synthesis and structural characterization of core-sheath hydrogel optical fibers by ILDWS

**Figure 1.**
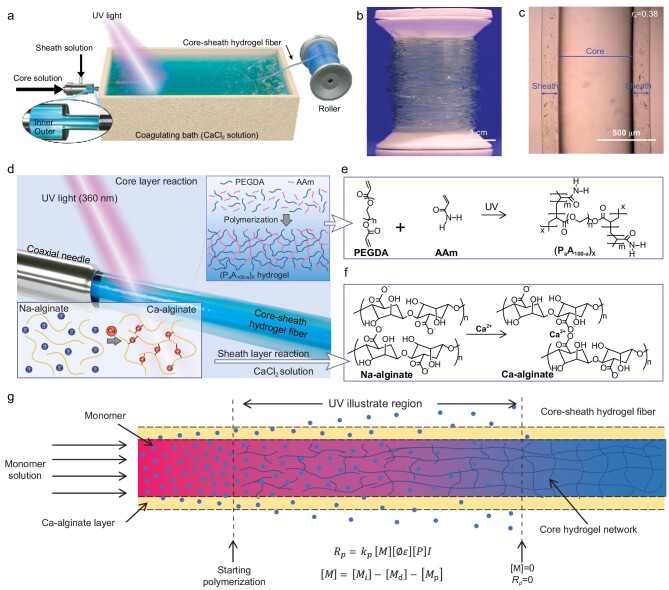
Synthesis of core-sheath hydrogel optical fibers via integrated dynamic wet spinning process (ILDWS). (a) Schematic illustration of the integrated dynamic wet spinning apparatus. (b) Photograph of a rolled core-sheath hydrogel fiber (the core was dyed with methylene blue for clarity of illustration). (c) Side-view optical image of a core-sheath hydrogel fiber (*r_e__ _*= 0.38). (d) Schematic illustration and chemical reactions for synthesizing core-sheath hydrogel fibers. Schematic formation of (e) core hydrogel and (f) sheath hydrogel. (g) Schematic illustration of formation mechanism of core-sheath hydrogel fiber.

The schematic representation of the ILDWS process is illustrated in Fig. 1a with fabrication details reported in the experimental section of the Supplementary Data. Briefly, the core spinning solution consisting of a PEGDA + AAm monomer mixture in deionized water [defined here as (P_a_A_100-a_)_X_, where a = mass% of PEGDA in the monomer mixture, 100-a = mass% of AAm in the mixture and X = total monomer concentration in wt% in the spinning solution] and a sheath spinning solution (Na-alginate aqueous solution) were extruded into a coagulating bath (CaCl_2_ solution, 1.5 wt%) through the self-designed core-sheath spinning needle. The sheath solution gelled in the CaCl_2_ solution by ionic crosslinking; simultaneously, the core solution was crosslinked by the initiator (I2959) under 360 nm UV light. In a typical ILDWS experiment, a fully crosslinked core-sheath hydrogel fiber as long as 10 m (a segment is shown in Supplementary Fig. 1) was synthesized continuously and collected onto a bobbin (Fig. 1b) at a winding speed of 30 cm min^−1^. Scale-up of the ILDWS process to produce kilometer-long fibers is easily envisioned. The chemistry, optimization of the fiber optical quality by varying processing parameters (monomer weight ratios, extrusion rates, see Table 1 and Supplementary Tables 1–3) and the corresponding microstructure evolution are discussed in detail below (*vide infra*). The achievement of a distinct and uniform core-sheath fiber structure was evidenced by cross-section scanning electron microscopy (SEM) (Supplementary Fig. 2) and side-view optical images (Fig. 1c and Supplementary Fig. 3a).

**Table 1.** Summary on the dimension, tensile properties and light attenuation of (P_50_A_50_)_40_ optical waveguide hydrogel fibers (15-cm length) fabricated by different *r_e_*. *M_n_* of the PEGDA was 700.

*r_e_*	Extrusion rate of core solution (cm min^−1^)	Diameter (core/sheath, μm)	Attention with 450 nm laser (dB cm^−1^)	Attention with 515 nm laser (dB cm^−1^)	Attention with 650 nm laser (dB cm^−1^)	Tensile strength (MPa)	Young's modulus (MPa)
0.25	60	841 ± 56/1172 ± 69	0.46 ± 0.03	0.20 ± 0.01	0.18 ± 0.01	0.79 ± 0.03	2.58 ± 0.09
0.38	40	667 ± 48/950 ± 72	0.53 ± 0.02	0.23 ± 0.02	0.27 ± 0.01	0.76 ± 0.02	2.25 ± 0.11
0.50	30	541 ± 37/845 ± 42	0.49 ± 0.02	0.26 ± 0.01	0.28 ± 0.02	0.74 ± 0.03	2.12 ± 0.08
1.00	15	411 ± 35/859 ± 41	0.57 ± 0.03	0.38 ± 0.02	0.30 ± 0.01	0.58 ± 0.02	1.68 ± 0.10
1.50	10	339 ± 26/776 ± 44	0.56 ± 0.03	0.54 ± 0.05	0.35 ± 0.02	0.44 ± 0.02	1.37 ± 0.14
2.00	7.5	276 ± 33/780 ± 77	0.59 ± 0.04	0.63 ± 0.06	0.38 ± 0.02	0.30 ± 0.03	0.61 ± 0.07

*r_e_* = extrusion rate of sheath solution: extrusion rate of core solution, where the extrusion rate of sheath solution was kept at 15 cm min^−1^.

To show the versatility of the dynamic wet-spinning concept, two core-sheath fiber systems with different chemical structures and rheology properties were fabricated successfully. As shown in Supplementary Figs 4 and 5, such like NIPAm*co*-DMAAm-based hydrogel (Supplementary Fig. 4a and b), (OEGMA-*co*-MEO_2_MA)/p(PEGDA-*co*-AAm)/Ca-alginate-based semi-interpenetrating hydrogel fibers (Supplementary Fig. 5a and b) could also be fabricated, which are similar to the OWHF based on p(PEGDA-*co*-AAm).

The key feature of ILDWS is that UV light is used to trigger polymerization, leading to the creation of the core-sheath structure. Figure 1d shows the details of the region around the needle and the UV irradiation zone. First, with the sheath and core solutions injected coaxially into the coagulating bath, the Ca-alginate sheath hydrogel fiber began to form immediately. The process involved the diffusion of Ca^2+^ ions into the sheath solution and subsequent crosslinking with alginate molecules [23], as shown in Fig. 1d-bottom and Fig. 1f. The gelled sheath layer then stabilized the core solution, allowing it to continuously flow into the UV irradiation region. This process was essential for the continuous synthesis of the core-sheath fiber without breaking points. Under UV irradiation, polymerization/crosslinking of PEGDA/AAm occurred by a radical polymerization to form the crosslinked network (Fig. 1d-top and Fig. 1e), which could be proven by the ^13^C nuclear magnetic resonance (NMR) spectra in Supplementary Fig. 6; in addition, almost no double bonds in the P_50_A_50_ hydrogel fiber were detected, indicating that the monomers including PEGDA and AAm were adequately converted into a polymer network. The formation of the core layer hydrogel fiber involved the balance between the monomer diffusion in the coagulating bath and the formation of a hydrogel network from the polymerization under the UV light, as shown in Fig. 1g. The degree of cross-linking of the hydrogel was mainly dependent on the rate of the polymerization process (*R_p_*), shown as follows [24,25]:

(1) \begin{equation*} {R_p} = {k_p}[M][\O \varepsilon ][P]I, \end{equation*}

where *k_p_*is the chain propagation rate constant*,* [*M*] is the monomer concentration, [Ø*ϵ*] is the efficiency of the initiator (Ø) and molar extinction coefficient (ϵ), [*P*] is the photoinitiator concentration and *I* is the UV light intensity.

Thus, when the core layer hydrogel fiber reached the place where *R_p_* = 0, the core layer hydrogel fiber was synthesized. This reaction was monitored by Fourier transforms infrared (FTIR) (Supplementary Fig. 7), where the intensity of the C=C peaks at 1610 and 980 cm^−1^ notably decreased upon UV irradiation [16]. In our systems, the crosslinked and randomized network structure affords a robust and stable fiber core as well as a uniform microstructure minimizing light loss, as experimentally demonstrated (*vide infra*). Further, fiber dimension, optical, morphological and mechanical properties could be controlled by monomer content parameters [a and X of (P_a_A_100-a_)_X_], the extruded solution viscosities and the ratio between the rate of extrusion of the core and that of the sheath solutions (*r_e_*). We anticipate that the most proper monomer precursor composition affording the optimized optical properties is a = 50 wt% and X = 40 wt% and the properties of (P_50_A_50_)_40_ core-sheath fibers with diameters tuned from 841 ± 56/1172 ± 69 to 276 ± 33/780 ± 77 (core diameter/sheath diameter, all units in μm) are reported in Table 1. From the data of Table 1 and Supplementary Fig. 3b, adjusting the spinning parameters (*r_e_*, from 0.25 to 2.00) can clearly afford fibers with different diameters (see details in Supplementary Figs 2 and 3) to suit different applications.

### Fiber microstructure design for optimal waveguiding properties

For optical fiber applications, the transparency of the core material and difference in the refractive index (*n*) between core and sheath materials can significantly influence light propagation properties. To optimize the desired waveguiding characteristics, the core material should possess a higher refractive index than the sheath material as well as maximum optical transparency at the wavelength of interest [7,10]. To this end, selection of the proper PEGDA molecular mass, optimization of the PEGDA : AAm content and spinning solution parameters are critical. Supplementary Fig. 8 demonstrates that the PEGDA molecular mass affording the best tradeoff between transparency and cost was 700 Da. To access transparency of the core material, we investigated the UV-visible spectral data for the hydrogel as a function of (P_a_A_100-a_)_X_ composition (see experimental section in the Supplementary Data). Thus, for PEGDA only solutions [(P_100_A_0_)_X_; a = 100%, X = 10–90 wt%, Supplementary Table 1], when the monomer concentration was below 60 wt%, the transmittance of polymerized PEGDA was less than 70% in the visible range (Supplementary Fig. 9a) as a result of polymerization-induced phase separation caused by the immiscibility of PEGDA with water [7]. Moreover, for compositions comprising AAm [(P_a_A_100-a_)_X_; a = 10–90% and X = 30 wt% (Supplementary Table 2), a = 50%, X = 20–90 wt% (Supplementary Table 3)], hydrogel transmittance improved significantly (Supplementary Fig. 9b). For instance, when the AAm content increased to 50% (in total monomers), hydrogel transmittance reached ∼90% in most of the visible region. Thus, P_50_A_50_ was chosen as the optimized monomer ratio for the studies on extruded fibers (Supplementary Fig. 9c). Supplementary Fig. 9d presents the refractive index of the chosen raw materials, where both core (P_50_A_50_)_X_ (X = 20–90 wt%) and sheath material (Na-alginate) exhibited a linear relationship with increasing concentration. Moreover, the refractive index (*n* = 1.352) of (P_50_A_50_)_10_ hydrogel with the minimum total monomer concentration was higher than that of Ca-alginate with the maximum concentration of 4% (*n* = 1.339). These properties meet the requirements for use in optical fiber applications [6,10].

As discussed previously, the rheological behavior of the solution can greatly affect the spinnability and stability during fiber fabrication [18,20] and thus can strongly influence morphological quality and light propagation through the resulting fiber. Supplementary Fig. 10a shows that all (P_50_A_50_)_X_ solutions exhibited Newtonian fluid behavior, where the viscosity did not change with increasing shear rate. In addition, the viscosity of the (P_50_A_50_)_X_ solutions was quite low, especially for (P_50_A_50_)_20_ solution, which exhibited a viscosity that was only ∼2 times higher than the viscosity of water (1.95 × 10^−3^ Pa s vs. 0.89 × 10^−3^ Pa s, 25°C, respectively). These data suggest that the (P_50_A_50_)_X_ solutions exhibit good fluidity, as confirmed by the (P_50_A_50_)_X_ solution images presented in Supplementary Fig. 10b. Further, the Na-alginate solutions also exhibited a steady liquid behavior (Supplementary Fig. 10c), so both the core and sheath solutions could enable the continuous fiber spinning process. We demonstrate that the proper selection of (P_50_A_50_)_40_ core composition and Na-alginate solution concentration (2 wt%) enabled the fabrication of high-quality core-sheath OWHF in a stable and continuous manner.

### Evaluation of light propagation properties

**Figure 2.**
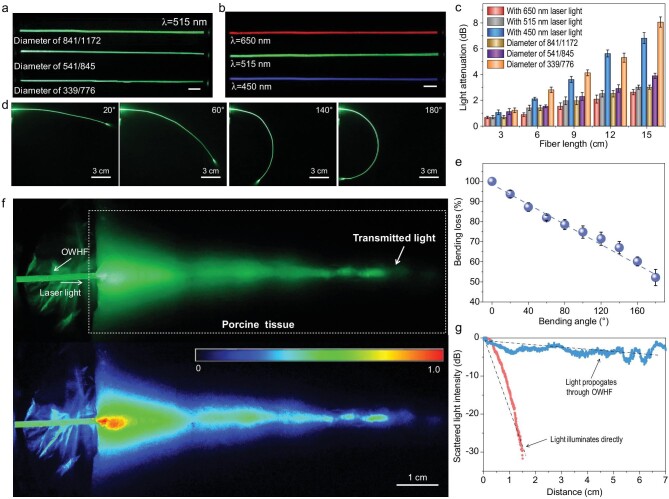
Light propagation for (P_50_A_50_)_40_ OWHF: light transmission within hydrogel fiber with (a) different fiber diameters (scale bar: 1 cm) and (b) different laser wavelengths (fiber diameter = 841/1172) (scale bar: 1 cm). (c) Light attenuation of scattered light along with the fiber profile. (d) Photographs and (e) bending loss of light propagation through OWHF with different bending angle. (f and g) Light transmission in porcine tissue with implanted OWHF (541/845).

Light propagation through the (P_50_A_50_)_40_ OWHF samples was assessed by focusing the laser light on one fiber tip and measuring the scattered light intensity over the hydrogel fiber lengths (15 cm). Supplementary Fig. 11 provides a comparison of light propagation through the core hydrogel fibers in the absence of a sheath (control fiber) and the OWHF. While the control fiber exhibited severe scattering with high light loss, the OWHF showed efficient light propagation due to effective reflections at the core-sheath interface [9]. Light propagation tests in the visible region were carried out using red (λ = 650 nm), green (λ = 515 nm) and blue (λ = 450 nm) laser light sources. Figure 2a and b and Supplementary Fig. 12 show that each visible wavelength efficiently propagated through the OWHF, and that transmitted light intensity, could be controlled by adjusting the laser power. Analysis of the intensity profile of the scattered illumination is presented in Fig. 2c. As the fiber core/sheath diameters increased from 276 ± 33/780 ± 77 to 841 ± 56/1172 ± 69, green light (λ = 515 nm) attenuation decreased from 0.63 ± 0.06 to 0.20 ± 0.01 dB cm^−1^. When the diameter of the hydrogel fiber was larger, light propagated over a longer distance before being reflected at the core-sheath interface. Furthermore, as seen in Fig. 2c and Table 1, when the wavelength increased from 450 to 650 nm, light attenuation decreased. For example, attenuation through 15 cm of fiber with diameter = 841 ± 56/1172 ± 69 decreased from 0.46 ± 0.03 to 0.18 ± 0.01 dB cm^−1^ because the transmittance of the longer wavelength light was higher (Supplementary Fig. 9c), leading to lower attenuation, a phenomenon that has also been found in soft polymer optical fibers [9]. In a manner that is comparable to other systems such as cladded soft polymer optical fibers (0.1, 0.4 dB cm^−1^ with 532 and 491 nm laser light, respectively, the core fiber diameter = 1 mm) [9], Ca-alginate cladded hydrogel fiber (∼0.19 dB cm^−1^ with 532 nm laser light, the core fiber diameter = 2 mm) [7], step-index optical fiber made of biocompatible hydrogels (0.32 dB cm^−1^ with 492 nm laser light, fiber diameter = 800/900 μm) [10], strain-sensing hydrogel optical fibers (0.45 dB cm^−1^ with 532 nm laser light, fiber diameter = 750/1100 μm) [3], and alginate-PAAm hydrogel fiber (0.25 dB cm^−1^ with 472 nm laser light, fiber diameter = 500 μm) [6] are summarized in Supplementary Fig. 13. For biomedical *in vivo* applications, it is essential to consider the curvature of body parts and organs in the optimization of the optical signal and energy. Thus, the light propagation efficiency under different bending angles should be evaluated as well. The bending loss was tested by measuring the output light intensity at different bending angles of 0° to 180°. As the bending angle increased, the intensity of propagated light decreased by ∼50% at 180° (Fig. 2d and e). The bending losses in the current OWHF were probably due to the deformation of the sheath mantle as also seen for other systems [7,9]. In addition, benefitting from its soft properties, the OWHF was easily recovered after bending 180° and showed an excellent cyclic stability of light propagating through OWHF before bending and after bending 180° (Supplementary Fig. 14).

To investigate the suitability of our fiber for *in vivo* biological tissue applications, light propagation was first tested by sandwiching an (P_50_A_50_)_40_ OWHF (diameter = 541 ± 37/845 ± 845) between two gelatin matrices and illuminating with a 515 nm laser (0.5 mW) focused at one end. The top view of propagation through a fiber embedded within the gelatin matrix with/without laser illumination is provided in Supplementary Fig. 15a. Propagation of light was clearly apparent through the OWHF (attenuation <20%, Supplementary Fig. 15b), but hazy laser light was observed in the gelatin matrix surrounding the hydrogel fiber due to the light loss caused by contact between the OWHF and simulat tissue [7,9]. Furthermore, *in vivo* light propagation tests (Fig. 2f and g, and Supplementary Fig. 16a) in porcine tissue without/with implanted OWHF were also performed. In the absence of fiber, illuminating the porcine tissue surface led to light penetration to a depth of only 1.5 cm. Alternatively, with an implanted OWHF, light was observed to effectively propagate along the entire length of the implanted hydrogel fiber. In addition, due to light scattering at the air/tissue interface and within the tissue, the attenuation of scattered light intensity was 0.62 dB cm^−1^ (Fig. 2g). Furthermore, our OWHF could also be used as a kind of sewable optical thread to efficiently transport light through porcine tissue, as shown in Supplementary Fig. 16b.

### Mechanical properties and biocompatibility

**Figure 3.**
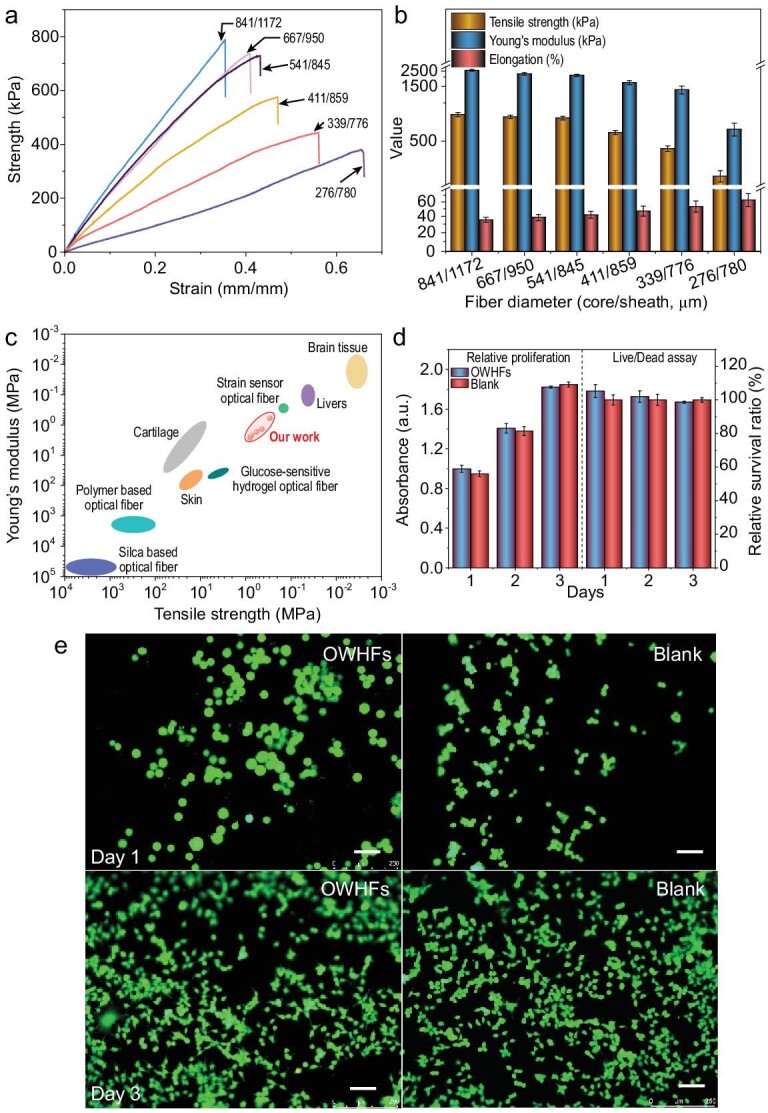
OWHF compatibility with tissues: (a) mechanical properties typical of tensile stress-strain curves, (b) strength, elongation and modulus of the OWHF dependent on the fiber diameters and (c) mechanical properties compared with other light-guide fibers. (d and e) Relative proliferation and live/dead assay of NIH-3T3 cells on OWHF compared to blank at 1 and 3 days, where live cells are in green, and dead cells in red. Scale bars: 100 μm.

For *in vivo* applications, particularly when implanted into the human brain/deep tissues, a waveguide fiber must be biocompatible and exhibit comparable mechanical properties to avoid serious and irreversible trauma, such as physical damage to the host tissue and inflammation [2,6,26,27]. Figure 3a and b presents typical tensile stress curves and a summary of the mechanical properties (strength, modulus and elongation) for (P_50_A_50_)_40_ OWHFs of different diameters, demonstrating that the Young's modulus/tensile strength decreased from 2.58 ± 0.09 MPa/0.79 ± 0.03 MPa to 0.61 ± 0.07 MPa/0.30 ± 0.03 MPa, while elongation increased from 35.5% to 63.0% when the fiber diameter decreased from 841 ± 56/1172 ± 69 to 276 ± 33/780 ± 77 (core diameter/sheath diameter). This is because, as shown before, by increasing *r_e_,* the fiber core diameter decreased rapidly, while the sheath diameter first decreased and then varied only slightly. The p(PEGDA/AAm) core material has been shown to exhibit a higher strength than the Ca-alginate sheath, and thus, it dominates the mechanical properties [17,23]. Figure 3c compares the tensile properties of several fibers demonstrating the superiority of OWHF versus typical polymer (PMMA, PS, PC) and silica-based optical fibers [28–32]. Tissues in the human body have soft and wet properties [30–33], and typical examples are presented in Fig. 3c: cartilage (Young's modulus of 30–100 MPa), skin (Young's modulus of 0.8–40 MPa), liver (Young's modulus of 0.05–0.25 MPa) and brain tissue (Young's modulus of 0.005–0.06 MPa). Thus, given the OWHF Young's modulus (0.61–2.58 MPa), we anticipate that it is suitable for *in vivo* applications, especially for delivering light signals within soft tissues. In addition, the OWHF in this study possesses a Young's modulus similar to that of strain-sensing hydrogel optical fibers [3] and only slightly lower values than glucose-sensitive hydrogel optical fibers [7]. Furthermore, due to its excellent mechanical properties, the results in Supplementary Fig. 17 show that the OWHF could suffer a long-term large body movement (model of sandwich between two pieces of porcine tissues, and repeated bending 180°) without breakdown of the OWHF.

As an implantable device, the OWHF is expected to have excellent biocompatibility, as demonstrated here through cell viability and live/dead viability assay tests. NIH-3T3 cells were used as a model cell and cultured with an (P_50_A_50_)_40_ OWHF in 96-well plates, and the results are given in Fig. 3d and e. No significant difference was observed between the blank and OWHF**-**containing samples, indicating that the hydrogel fiber did not suppress cell growth. In addition, the results of the live/dead assay test demonstrate that with time (increase in days of culture), the cell number increased (in this test, all cells were stained green, where the image confirmed the high relative cellular survival ratio). These results are in good agreement with prior studies using PEGDA composite materials [1,7]. Furthermore, the swelling behavior of this OWHF in deionized water was investigated, which demonstrated that the diameter of the core fiber stabilized at approximately 715 μm with time (approximately 1440 min) due to the high crosslink density, whereas the diameter of the fiber sheath increased only slightly from 1158 to 1368 μm (Supplementary Fig. 18). The results suggest that the OWHF is suitable for use in *in vivo* wet environments due to the stable swelling behavior.

### Deep-tissue photothermal therapy

**Figure 4.**
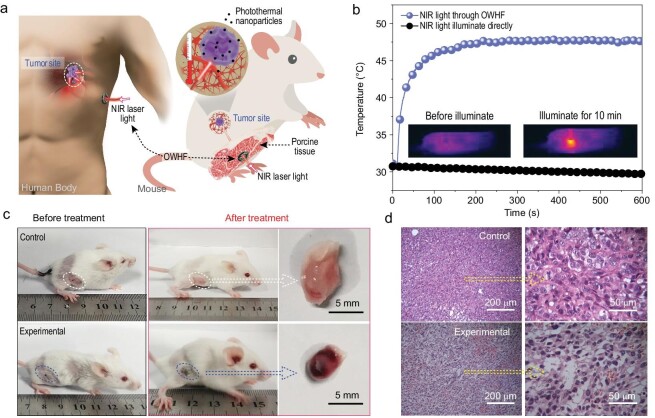
The deep-tissue photothermal therapy: (a) model of *in vivo* cancer therapy in which the NIR light was transported through OWHF. (b) Temperature change and infrared (IR) thermal images (inset in b) of muscle and tumor after exposure under NIR light which was transported through OWHF. (c) Photos of mice before and after cancer therapy. (d) H&E stained tumor slices collected from control and experimental groups.

Even though laser-aided photothermal therapy has been widely utilized for cancer therapy in the past two decades [34–36], there are still considerable limitations for subcutaneous deep-tissue applications, mainly due to limited, centimeter-scale light penetration through skin tissues. In recent years, the use of optical fibers for *in vivo* biomedicine has received substantial attention in applications such as photodynamic therapy [37,38] and biomedical sensing [10,39]. Among these applications, conventional optical fibers (including silica- and polymer-based fibers) serve as the standard tools for optical implants. However, their use often incurs problems due to the inferior compatibility and mechanical stiffness that can cause inflammation and physical damage to the host tissue. The OWHF fabricated in this study possess excellent biocompatibility, tissue-like mechanical properties, good light propagation properties and superior processability allowing for continuous fabrication, so they may be suitable for deep-tissue photothermal therapy. A possible *in vivo* application related to the delivery of light to a tumor site through a complex path is illustrated in Fig. 4a. The deep-tissue photothermal cancer therapy model involving the application of our OWHF is presented on the right of Fig. 4a, and a simple illustration of the homemade device used for the test is provided in Supplementary Fig. 19c. Porcine tissue, which is often used as a human-tissue model, was utilized in our study to simulate human tissue [7,9]. Briefly, a (P_50_A_50_)_40_ OWHF (diameter of core/sheath = 1.8 mm/2.2 mm) was implanted in porcine tissue (∼5 cm thick) and positioned near the tumor site of a mouse. The tumors were first intratumorally injected with a CuS dispersion (dispersed in normal saline). Two hours post-injection, the tumor was irradiated with a 915 nm near infrared (NIR) Laser via the OWHF, upon which the temperature of the tumor site was found to rapidly increase to 48°C. In contrast, NIR illumination directly through porcine tissue led to no measurable temperature increase at the tumor site (Fig. 4b). These results confirmed that the hydrogel fiber could serve as an effective vehicle to deliver the NIR light to deep tissue. After several therapy sessions, tumors in the experimental mouse group were notably smaller and darker red than those in the control group that did not receive treatment (Fig. 4c). These results demonstrate effective cancer therapy using NIR laser therapy through an OWHF based on the photothermal effect. To further verify the antitumor efficiency, hematoxylin and eosin (H&E) stained tumor slices were prepared, and the results are presented in Fig. 4d. This figure shows that the cancer cells were severely damaged in the experimental group, while the blank control group exhibited no damage. Thus, the OWHF may serve as a viable and efficient tool for versatile deep-tissue therapy.

### Optogenetic stimulation for brain-interface communications

**Figure 5.**
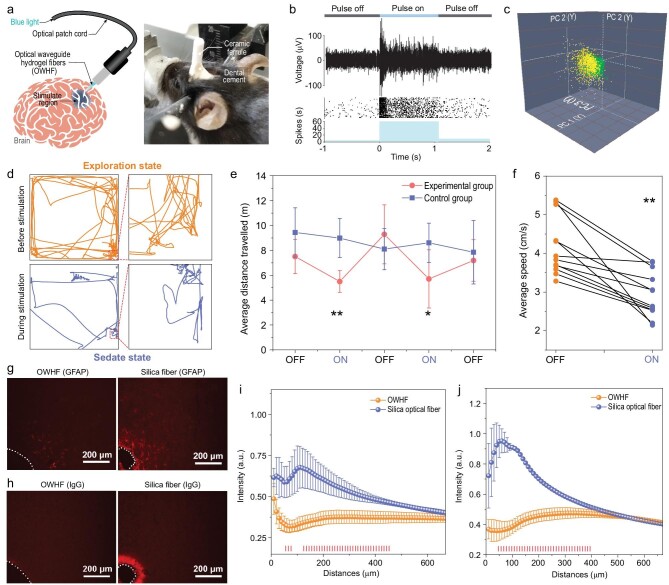
Optogenetic stimulation for brain-interface communications: (a) left part is schematic illustration of optogenetic stimulation and right part is the photo of a mouse implanted with an OWHF into M1 of the brain. (b) Electrophysiological test before and during optogenetic stimulation (example of raw trace, raster plot and peri-stimulus time histograms (PSTH) of recorded PV : ChR2 neurons). (c) The principal-component analysis of two separable neuronal units presented in (b). (d) Typical motion trail before and during optogenetic stimulation. (e) Traveled distance in the open field. (f) Average speed of mice before and during optogenetic stimulation (*n* = 6 mice, ^*^P < 0.05, ^**^P < 0.005, t-test). (g–j) Immune responses and blood-brain barrier function in OWHF and silica fiber implanted cortical tissue four weeks after the implant surgery: (g and i) GFAP and (h and j) IgG immunostaining of OWHF compared to conventional silica after implanted for four weeks. Red bars in (i) and (j) indicate statistically significant data points.

In the past decade, optogenetics has been widely used in neural circuit dissection as well as potential clinical applications for treating neurological disorders such as epilepsy [40], Parkinson's disease [41] and other brain disorders [26,42]. By expressing light-gated ion channels such as channelrhodopsin-2 (ChR2) [6,26,43], one can use light to control activities of genetically targeted neurons in a timely manner. One key component of *in vivo* optogenetic stimulation is the introduction of visible light into brain tissue using traditional optical fibers. However, the large difference between the Young's modulus of traditional optical fibers and the brain (∼10^4^–10^6^ difference, *vide supra*) could cause damage or inflammation [44]. Since the OWHFs discussed here are highly biocompatible with brain tissue-like Young's modulus (<2.60 MPa) and effective light propagation properties (0.63–0.18 dB cm^−1^ within the visible region at a length of 15 cm, *vide supra*), they may offer opportunities for the development of improved optical waveguides for stimulating regions in the brain, as shown in Fig. 5a.

To investigate the possibility of using our OWHF for optogenetic stimulation, we first tested whether the intensity of propagated light through our fiber is sufficient for optogenetic stimulation. Thus, a multi-electrode array was implanted into the primary visual cortex (V1) of parvalbumin-channelrhodopsin-2 (PV-ChR2) mice, with ChR2 expression in PV inhibitory neurons (Supplementary Fig. 20a and b). Blue light pulses were propagated into V1 through a (P_50_A_50_)_40_ OWHF (diameter = 339 ± 26/776 ± 44), and as shown in Fig. 5b, light-triggered spiking activity from one neuron was observed, suggesting that optogenetic activation of PV neurons resulted in the suppression of spiking activities in pyramidal neurons. In addition, the three-dimensional graph of the united clusters in Fig. 5c shows spiking activities from two different neurons as stimulated by the blue pulse, implying efficient light propagation into the brain tissue via the OWHF [6].

Finally, the ability of the OWHF to enable optogenetic stimulation *in vivo* was also explored. The OWHF was first coupled into a ceramic ferrule (as shown in Supplementary Fig. 20c) and then implanted into the primary motor cortex (M1) of PV-ChR2 mice (Supplementary Fig. 20a and Fig. 5a) with ChR2 expression in the PV inhibitory neurons (Supplementary Fig. 21a and b). The mice were allowed to freely explore an open-field environment before optical stimulation. When the light was on, the mice were visibly more sedated, less interested in exploring their environment and traveled a smaller distance (Fig. 5d and Supplementary Fig. 22). The average distance traveled and running speed decreased during optical stimulation, as shown in Fig. 5e and f, probably because activities in M1 are suppressed upon OWHF mediated optogenetic activation of PV neurons. Hence, as a biocompatible light-guide material, the OWHF may be suitable for optogenetic stimulation, exhibiting significant potential for optogenetic-based neuronal intervention.

In addition, we evaluated the immune responses and blood-brain barrier function as well as neuron density four weeks after the OWHF and silica fiber implant surgery. Astrocytes, immune-reactive cells labeled by glial fibrillary acidic protein (GFAP), were shown to be populated more densely near the silica fiber implant than the OWHF implant (Fig. 5g and i), indicating that the silica fiber implant triggered a more severe immune response than the OWHF implant [6,45]. In the OWHF**-**implanted cortical tissue, IgG, a common serum antibody in blood circulation [46], was restricted to the vasculature and removed upon perfusion (Fig. 5h). However, in silica fiber-implanted cortical tissue, a high level of IgG was detected (Fig. 5j), showing the dysfunction of the blood-brain barrier near the implant. We also found that microglia/macrophage activation, identified by CD68 immunohistochemistry, was more significant in the silica fiber implant than in the OWHF implant (Supplementary Fig. 23a and b). In addition, the neuronal density near the silica fiber was lower than that near the OWHF (Supplementary Fig. 23c and d). Together, these pieces of evidence suggested that the OWHF has better long-term mechanical interaction and biocompatibility with brain tissue than the silica fiber.

## CONCLUSION

In summary, a versatile design concept of ILDWS for the continuous production of core-sheath hydrogel optical fibers was demonstrated, where ILDWS allowed fine-tuning of the fiber diameter, as well as mechanical and optical-propagation properties. The wet spinning process was optimized through the rational design of the core/sheath material interface, optical transparency, refractive index and spinning solution viscosity. The best performing hydrogel optical fibers exhibited low optical attenuation (0.18 ± 0.01 dB cm^−1^ at 650 nm, distance of 15 cm), excellent biocompatibility and tissue-like Young's modulus (<2.60 MPa). In addition, the OWHF can serve as efficient light-guide fibers for the delivery of laser signals and energy, which is suitable for deep-tissue photomedicine such as photothermal therapy deep into the organism and optogenetic stimulation for brain-interface communications. Furthermore, the OWHF has better long-term mechanical interaction and biocompatibility with tissues than the silica fiber. Thus, the continuous wet spinning of optical waveguide hydrogel fibers can enable these materials to be broadly applied in photomedicine, optical biosensing and optogenetic stimulation.

## METHODS

See details in the Supplementary Data.

## Supplementary Material

nwaa209_Supplemental_File
