# Supplementary material for: Integrated dynamic wet spinning of core-sheath hydrogel fibers for optical-to-brain/tissue communications
Source: Natl Sci Rev. 2020 Aug 31;8(9):nwaa209. doi: 10.1093/nsr/nwaa209 (PMC8433079; doi:10.1093/nsr/nwaa209)
Supplement: nwaa209_Supplemental_File [file nwaa209_supplemental_file.docx]

**Supplementary Information**

**Integrated** **Dynamic Wet Spinning of Core-Sheath Hydrogel Fibers for** **Optical-to-Brain/Tissue Communications**

Guoyin Chen, ^#^ Gang Wang, ^#^ Xinrong Tan, Kai Hou, Qingshuo Meng, Peng Zhao, Shun Wang, Jiayi Zhang, Zhan Zhou, Tao Chen, Yanhua Cheng, Benjamin S. Hsiao, Elsa Reichmanis, Meifang Zhu*

^#^ Equal Contributions

^*^ To whom correspondence be addressed. E-mail: M.F. Zhu (zhumf@dhu.edu.cn)

Supplementary Data

1. **Experimental Section**

**Materials.** Sodium alginate (Na-alginate), gelatin, and acrylamide (AAm) were purchased from Sinopharm Chemical Reagent Co., Ltd. PEGDA (*M_n_* = 575 Da, 700 Da, 1000 Da), Oligo(ethylene glycol) methacrylate (OEGMA, *M_n_* = 475 Da), 2-(2-methoxyethoxy) ethyl methacrylate (MEO_2_MA, *M_n_* = 188 Da), N-Isopropyl acrylamide (NIPAm), N,N-Dimethylacrylamide (DMAAm) and photoinitiator 2-hydroxy-4’-(2-hydroxyethoxy)-2-methylpropiophenone (IRGACURE 2959, I2959, 98%) were supplied by Sigma-Aldrich. Calcium chloride anhydrous was purchased from Shanghai Lingfeng Chemical Reagent Co., Ltd. Deionized water was supplied by a water purification system (Heal Force Bio-Meditech Holdings Ltd.).

**Selection of** **PEGDA monomer and transmittance optimization of crosslinked PEGDA films.** As shown in Supplementary Fig. 8, it can be seen that with increasing *M_n_*, the PEGDA hydrogels became more transparent due to the higher degree of hydrophilicity of PEGDA. Furthermore, considering the cost of PEGDA (the higher *M_n_*, the higher cost), the PEGDA with *M_n_* = 700 Da was chosen as the raw material. PEGDA and I2959 (PEGDA : I2959 = 1000 : 5, wt%) were added into deionized water under magnetic stirring in the dark environment to prepare PEGDA solutions at different concentrations (10 wt% - 90 wt%). Different amounts of acrylamide (AAm) were added into the PEGDA solution to optimize the transmittance of the PEGDA gel, where the detailed pre-gel dispersions of the transmittance optimization process with different compositions are shown in Tables S1- S3. In this optimization process, the sample was defined here as (P_a_A_100-a_)_X_, where a = mass % of PEGDA in the mixture, 100-a = mass % of AAm in the mixture monomers, and X = total monomer concentration in wt.% in the spinning solution.

**Preparation of core-sheath hydrogel fiber.** (P_50_A_50_)_40_ monomer solution and Na-alginate solution (2.0 wt%) were chosen as the core and sheath solutions, respectively, to fabricate the core-sheath hydrogel optical fiber. These two solutions were injected into the coagulating bath (CaCl_2_ solution at 1.5 wt% concentration, size of 18 cm in length, 8 cm in width, and 5 cm in high) through a coaxial needle (the sheath diameter was 1.469 mm and the core diameter was 0.788 mm) by two metering pumps (KDS100, KD Scientific, USA) simultaneously. The extrusion rate ratio (*r_e_*, the adjusting range was 0.25 - 2.00, the sheath extrusion rate was immutable at 15 cm/min) of sheath:core was controlled to adjust the diameter of the core-sheath fiber. In addition, the mercury lamp UV irradiation (S1500, EXFO, Canada. λ = 360 nm, 2.77 W/cm^2^) was placed in the water bath to initiate the photopolymerization of the extruded core monomer solution. The fabricated core-sheath hydrogel fiber was collected on a roller outside the water bath, and the winding speed was set at 30 cm/min. The schematic illustration of the spinning process was illustrated in Fig. 1a. Finally, the obtained core-sheath fibers were exposed under the UV irradiation (λ = 360 nm, 2.77 W/cm^2^) for 30 min and then immersed in deionized water for three days.

**The versatility of the fabrication concept**. To prove the versatility of the dynamic wet-spinning concept, two core-sheath fiber systems with different chemical structure and rheology property were fabricated successfully. Briefly, NIPAm-*co*-DMAAm based core-sheath hydrogel fiber was prepared according to the same method with the (P_50_A_50_)_40_ core-sheath hydrogel fiber; (OEGMA-*co*-MEO_2_MA)/p(PEGDA-*co*-AAm)/Ca-alginate based semi-interpenetrating hydrogel fibers was prepared by first synthesis the OEGMA-*co*-MEO_2_MA copolymer, then mixed with PEGDA and AAm monomer, finally the semi-interpenetrating hydrogel fibers was fabricated through the same method.

**Material characterization.** The optical transmittance of PEGDA and PEGDA/AAm hydrogels were characterized on a TU1901 UV-vis spectrometer (Purkinje General) in the wavelength interval between 400 and 700 nm, which the hydrogels were gelled in a polymer cube (1×1×3, length×width×high) with a 360 nm UV light. Refractive index (RI) of the raw materials was measured by an Abbe refractometer (Shanghai CSOIF Co., Ltd.). The rheology property of the pre-gel dispersion was tested by a rotational rheometer (ARES, TA, USA) at 25 ^o^C, all the measurements were carried out using a concentric cylinder under the steady-state scanning shear rate of 0.01 - 1000^-1^ (outside cup diameter 34.0 mm, bob diameter 32.0 mm, bob height 33.0 mm, immersed height 53 mm, and bottom gap 2000 μm). Cross-section morphologies of the freezing dried core-sheath fibers were investigated by the field emission scanning electron microscopy (FE-SEM, S4800, Hitachi, Japan). Fiber diameters were carried out by stereoscopic microscopy (SMZ745T, Nikon, Japan). Fourier transforms infrared (FTIR) spectra were obtained by a Fourier transform infrared spectrophotometer with wavenumber ranged from 4000 to 600 cm^-1^ (Nicolet 6700, Thermo Fisher, USA). The mechanical properties of the hydrogel light-guide fibers were evaluated on an XQ-1A fiber tension tester (Shanghai New Fiber Instrument, China): The gauge length was 10 mm and the extension rate was 2 mm min^-1^. At least 10 filaments of each fiber were tested for the mechanical test, and the average values of strength, modulus, elongation were calculated based on the average cross-sectional area of the fibers.

**Light propagation test.** In this test, laser light (λ=450, 515, 650, 915 nm) was focused on one tip of the core-sheath hydrogel fiber, where the laser light intensity was measured by a handheld optical power meter (Newport, 1919R). The light transmission of the core-sheath hydrogel optical fiber in simulated tissues was tested by using gelatin and porcine tissue as the model. Photographs of the core-sheath hydrogel fiber propagate laser light were taken by a digital camera (Canon, EOS 80D) using a lens (Canon Macro EF 100 mm 1:2.8 L IS USM) operated at 1/1600 speed, ISO 1600, and F3.5. The obtained photographs of the core-sheath hydrogel fiber equipped with laser light were analyzed by using software Image J according to other works reported [S1-4]. Bending loss and bending cyclic stability for light propagating through OWHF was tested by changing the fiber bending angle (0 ^o^ - 180 ^o^) based on the measurement of the light intensity through another tip.

**Cell viability assay.** NIH-3T3 cells were seeded on 96-well plates with a density of around 10,000 cells per well, where the cells were allowed to adhere for 12 h prior to the assays. The cells were co-incubated with a series of core fiber and core-sheath hydrogel fiber for 1 - 3 days, where10 uL CCK8 were subsequently added to each well. The cells were then incubated for another 2 h at 37 ^o^C in the dark. The absorbance of CCK8 at 450 nm was measured by using a microplate reader (Bio-Rad, USA) to determine the cell viability. The values obtained were expressed as a percentage of the control cells to which no sample was introduced.

**Live/dead viability assay.** NIH-3T3 cells were seeded on 24 well glass bottom plates (Corning, USA) with a density of 10000 cells per well and allowed to adhere for 12 h prior to the assays. After 1, 2 and 3 days of treatments in a cell culture, the spent media was aspirated. The cells were rinsed twice with PBS, and subsequently added with 200 μL of staining solution (Calcein-AM/Propidium iodide, PI) in the following manner. Calcein-AM was first added to the sample and then incubated for 15 min at 4 ^o^C. The sample was then washed with PBS and added with PI to dye for 5 min. The resulting sample was then imaged by a fluorescence microscope (LEICA DMi8, Germany).

***In vivo* photothermal cancer therapy.** All the animal experiments were carried out according to the guidelines for the institutional committee for animal use and care regulation. CuS nanoparticles were synthesized according to a previous report [S5]. BALB/c Mice was obtained from Shanghai SLAC Laboratory Animal Center (Shanghai, China). In this study, 4T1 cells about 1×10^6^ were injected into backside of each mouse to prepare tumor-bearing mice. In specific, mice in the experimental group were injected with 100 μL CuS dispersions (in normal saline, 0.34 mg/mL), while mice in the control group were without treatment. For the *in vivo* photothermal cancer therapy, optical-waveguide hydrogel fiber (diameter = 1.8 mm/2.2 mm) was coupling with a 915 nm NIR laser light, and the intensity of NIR laser light was controlled at 0.35 W which was calibrated by a handheld optical power meter (Newport, 1919R). Tumor from the experiment group and the blank group were exposed to the 915 nm NIR laser light for 15 min (temperature was controlled at 48 ^o^C), which the NIR laser light was transported through the OWHF implanted into the porcine tissue as shown in Fig. 4a. The temperature change was recorded by using the photothermal therapy-monitoring system FLIR-A300 (FLIR Systems Inc., USA). A simplified illustration of the homemade photothermal therapy-monitoring device was shown in Supplementary Fig. 19c. Finally, mice were sacrificed and tumors were taken out and fixed in 10% formaldehyde solution for pathological section. The specimens were imaged by an optical microscope (VHM2600, VIHENT, China).

**Animal for optogenetic modulation.** Parvalbumin-channelrhodopsin-2 (PV-ChR2) mice were obtained by breeding PV-Cre mice (Jackson lab stock number: 008069) with flox-ChR2 mice (Jackson lab stock number: 012567). All animal care and protocols were performed in accordance with the National Institutes of Health Guide for the Care and Use of Laboratory Animals and were approved by the Animal Care and Use Committee at Shanghai Medical College of Fudan University.

**Recording of** **electrophysiological activities.** An optical-waveguide hydrogel fiber was first coupled to a blue laser light (472 nm, 10 mW, 0.2 Hz, 1 s duration) on one end, then coupled with the electrode (A 16 channel (4×4) platinum-iridium microelectrode array (1 MΩ, 250 μm apart, Microprobes, USA)) to be implanted into the primary visual cortex (V1) of mouse brain (anteroposterior -3.08 mm from bregma, mediolateral +2.5 mm, and dorsoventral -0.8 mm) as illustrate in Supplementary Fig. 20a and b. Single-neuron-level spiking activities were recorded using Cheetah (Digital Lynx, Neuralynx Inc, USA) under laser light stimulation.

**Optogenetic modulation of mouse behavior in open-field experiments.** To investigate the motor behavior of mouse under optogenetic stimulation, we conducted open-field experiments (40 cm × 40 cm) using PV-ChR2 mice. The core-sheath hydrogel fiber (diameter = 339/776) was first coupled into a ceramic ferrule (external diameter of 2.5 mm, inner diameter of 430 μm, as shown in Supplementary Fig. 21c), and then implanted into the primary motor cortex (M1, anteroposterior +1.92 mm, mediolateral 1.75 mm, and DV 1.46 mm) of PV-ChR2 mice as seen in Supplementary Fig. 21a and c. The fiber was secured to the skull with Metabond (Sun Medical Company, Ltd., Japan). A plastic cap was used to cover the ferrule to keep the hydrogel fiber moist prior to use. The mice were allowed to recover for three days before behavioral test. For the open-field experiment, mice of control group (six mice without core-sheath hydrogel implanted) and experimental group (PV-ChR2 mice with core-sheath hydrogel implanted) were allowed to move freely in an open field (40 cm × 40 cm) for 15 min (divided into five 3-min epochs). The blue light (472 nm, 10 mW, 20 Hz, 10 ms duration) was turned on in the experimental group during the second and fourth epoch. The entire process of open field experiment was recorded with DanioVision^TM^ observation chamber (Noldus Inc., Wageningen, The Netherlands) and analyzed by EthoVision XT 12 (Noldus Inc., Wageningen, The Netherlands).

**Immunohistochemistry.** The mice were anesthetized deeply and perfused with physiological saline *via* an aortic root catheter until the liver appeared to be white, followed by 4% paraformaldehyde (PFA). The brains were removed and post-fixed in 4% PFA overnight at 4 ^o^C, and then dehydrated in 30% sucrose until the sample sunk to the bottom. After immersing in cryo-embedding medium, brains were sectioned into 30-μm [horizontal](javascript:;) slices using a cryotome (Leica Microsystems, Wetzlar, Germany). Slices were first washed with Tris-buffered saline (TBS) for 5 times (5 min each), and permeabilized in 0.5% Triton-X-100 for 30 min, and then blocked with 3% bovine serum albumin for 2h. Slices were incubated overnight at 4 ^o^C with primary antibodies: anti-GFAP (SIGMA), anti-NeuN (SIGMA), anti-CD68 (Abcam) and anti-IgG (Abcam). The next day, the slices were washed for 5 times, and secondary antibodies (Jackson ImmunoResearch Laboratories) were applied to the slices and incubated at room temperature for approximately 2 h in the dark. The slices were washed for 5 times. Finally, slides were covered with coverslips and scanned randomly under a fluorescence microscope (Nikon, A1R, Japan) by a single investigator who was blind to sample identity. Quantification and statistical analysis of the obtained fluorescence image was performed using a software Python (version of 3.7.3). CV2 packet in Python were used to identify the location of the fiber, and then the average intensity of each image was calculated. The image was divided with a radius of 10 microns centered on the optical fiber. Finally, the average intensity for each division was calculated and analyzed using t-test in python.

1. **Continuous Synthesis and Structural Characterization of Core-Sheath Hydrogel Optical Fibers by ILDWS**

**Supplementary Table 1.** Detailed pre-gel dispersions/hydrogels of transmittance optimization with different PEGDA concentration.

| **Solution/Samples** | **PEGDA (g)** |  | **AAm (g)** | **I2959 (g)** | **Water (g)** | **Transmittance of hydrogel at 515 nm (%)** |
| --- | --- | --- | --- | --- | --- | --- |
| (P_100_A_0_)_10_ | 2.0 |  | 0 | 0.01 | 18.0 | 4.8 |
| (P_100_A_0_)_20_ | 4.0 |  | 0 | 0.02 | 16.0 | 4.3 |
| (P_100_A_0_)_30_ | 6.0 |  | 0 | 0.03 | 14.0 | 1.6 |
| (P_100_A_0_)_40_ | 8.0 |  | 0 | 0.04 | 12.0 | 24.6 |
| (P_100_A_0_)_50_ | 10.0 |  | 0 | 0.05 | 10.0 | 42.9 |
| (P_100_A_0_)_60_ | 12.0 |  | 0 | 0.06 | 8.0 | 57.8 |
| (P_100_A_0_)_70_ | 14.0 |  | 0 | 0.07 | 6.0 | 89.6 |
| (P_100_A_0_)_80_ | 16.0 |  | 0 | 0.08 | 4.0 | 91.0 |
| (P_100_A_0_)_90_ | 18.0 |  | 0 | 0.09 | 2.0 | 94.1 |

**Supplementary Table 2.** Detailed pre-gel dispersions/hydrogel of transmittance optimization with different monomer ratio of PEGDA and AAm under the same total monomer concentration.

| **Solution/Samples** | **PEGDA (g)** | **AAm (g)** | **I2959 (g)** | **Water (g)** | **Transmittance of hydrogel at 515 nm (%)** |
| --- | --- | --- | --- | --- | --- |
| (P_10_A_90_)_30_ | 0.6 | 5.4 | 0.03 | 14.0 | 99.0 |
| (P_20_A_80_)_30_ | 1.2 | 4.8 | 0.03 | 14.0 | 97.7 |
| (P_30_A_70_)_30_ | 1.8 | 4.2 | 0.03 | 14.0 | 97.5 |
| (P_40_A_60_)_30_ | 2.4 | 3.6 | 0.03 | 14.0 | 95.1 |
| (P_50_A_50_)_30_ | 3.0 | 3.0 | 0.03 | 14.0 | 94.8 |
| (P_60_A_40_)_30_ | 3.6 | 2.4 | 0.03 | 14.0 | 89.8 |
| (P_70_A_30_)_30_ | 4.2 | 1.8 | 0.03 | 14.0 | 80.8 |
| (P_80_A_20_)_30_ | 4.8 | 1.2 | 0.03 | 14.0 | 77.5 |
| (P_90_A_10_)_30_ | 5.4 | 0.6 | 0.03 | 14.0 | 69.3 |

**Supplementary Table 3.** Detailed pre-gel dispersions/hydrogel of transmittance optimization with different total monomer concentration of (P_50_A_50_)_X_.

| **Solution/Samples** | **PEGDA (g)** | **AAm (g)** | **I2959 (g)** | **Water (g)** | **Transmittance of hydrogel at 515 nm (%)** |
| --- | --- | --- | --- | --- | --- |
| (P_50_A_50_)_20_ | 2.0 | 2.0 | 0.02 | 16.0 | 82.8 |
| (P_50_A_50_)_30_ | 3.0 | 3.0 | 0.03 | 14.0 | 94.8 |
| (P_50_A_50_)_40_ | 4.0 | 4.0 | 0.04 | 12.0 | 95.7 |
| (P_50_A_50_)_50_ | 5.0 | 5.0 | 0.05 | 10.0 | 93.8 |
| (P_50_A_50_)_60_ | 6.0 | 6.0 | 0.06 | 8.0 | 94.6 |
| (P_50_A_50_)_70_ | 7.0 | 7.0 | 0.07 | 6.0 | 93.1 |
| (P_50_A_50_)_80_ | 8.0 | 8.0 | 0.08 | 4.0 | 96.5 |
| (P_50_A_50_)_90_ | 9.0 | 9.0 | 0.09 | 2.0 | 96.1 |


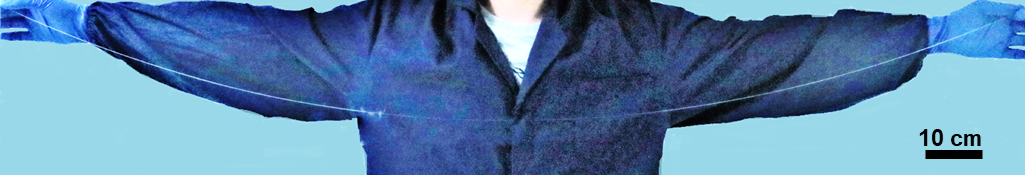


**Supplementary Figure 1.** Photograph of a segment of a (P_50_A_50_)_40_ core-sheath hydrogel fiber with a diameter of 541/845 (core diameter/sheath diameter).


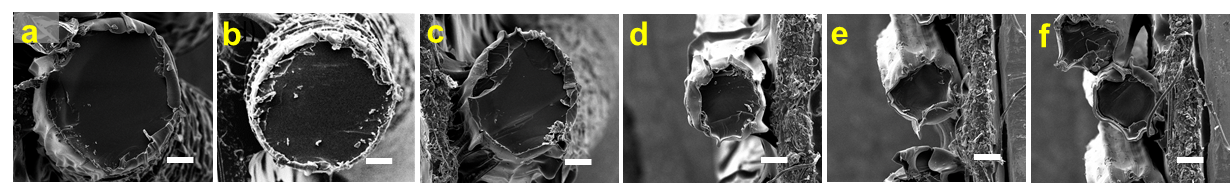


**Supplementary Figure 2.** Cross-sectional morphologies of (P_50_A_50_)_40_ core-sheath hydrogel fibers fabricated with (a) *r_e_* = 0.25, (b) *r_e_* = 0.38, (c) *r_e_* = 0.50, (d) *r_e_* = 1.00, (e) *r_e_* = 1.50, (f) *r_e_* = 2.00. Scale bars were 100 μm.


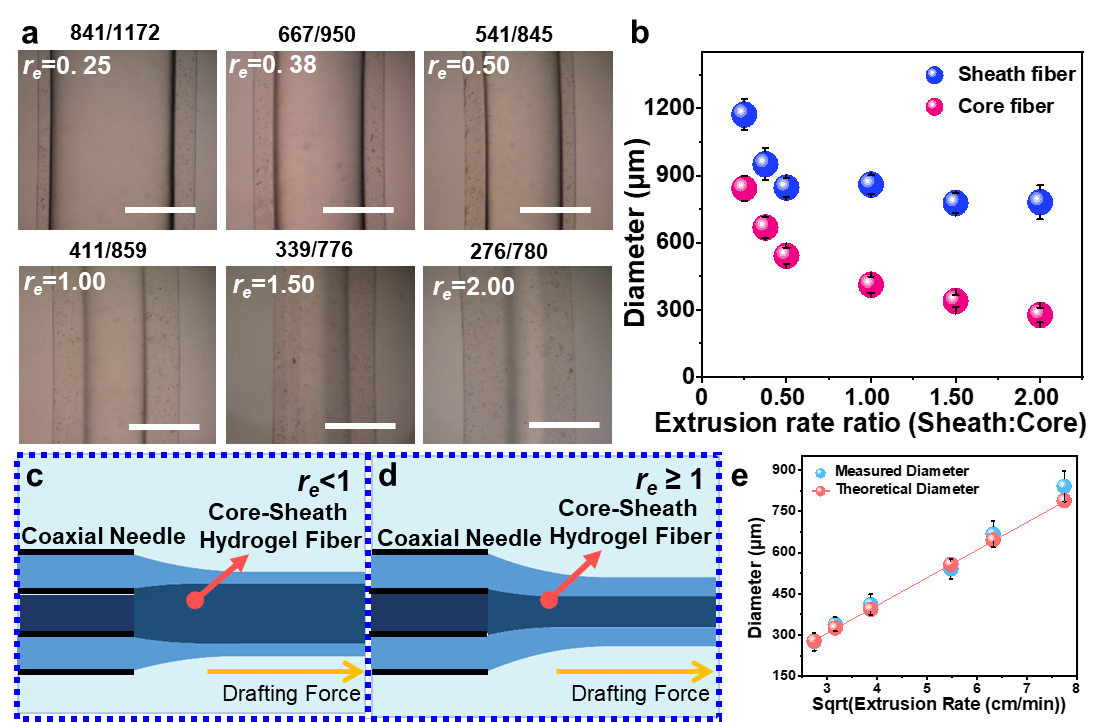


**Supplementary Figure 3.** (a) Photographs of What (P_50_A_50_)_40_ core-sheath hydrogel optical fibers based on different extrusion rate ratios (*r_e_*), scale bars are 500 μm. (b) Dependence of the diameters of the core-sheath hydrogel fibers on the extrusion ratio (*r_e_*, sheath : core). (c) and (d) schematic illustrations of the diameter control by different *r_e_*, (e) the measured core diameter compared to the theoretical diameter.

Supplementary Fig. 2 show the cross-section morphologies of freezing dried core-sheath hydrogel fibers under different *r_e_*. It is seen all the fibers possessed the core-sheath structure with a circular cross-section morphology, where the fiber diameters decrease with increasing the *r_e_* value under dry condition. In order to investigate the controllability of the core-sheath fiber diameter by this method, *r_e_* was adjusted by changing the extrusion rate of the core solution, and length of the core-sheath hydrogel fiber prepared under different *r_e_* (0.25, 0.38, 0.50, 1.00, 1.50 and 2.00) was in the meter range (Fig. 1b and Supplementary Fig. 1). Supplementary Fig. 3a shows the profile of core-sheath hydrogel fiber prepared under different *r_e_*. It is found that the diameters of these fibers decrease with increasing *r_e_*, a data consistent with the SEM results in Supplementary Fig. 2. The results from further measurements of the core and sheath diameter are shown in Supplementary Fig. 3b. It is seen that with increasing *r_e_*, the sheath diameter first decreases rapidly (from 1172 μm to 845 μm) and then more slowly (845 μm to 780 μm), while the core diameter decreases rapidly from 841.8 μm to 276.4 μm. As illustrated in Supplementary Fig. 3c, when *r_e_*<1, the extrusion rate of the core solution is higher than that of the sheath solution. Thus, under the constant winding speed, the core solution will expand, leading to the larger fiber diameter. In contrast, when *r_e_* ≥1 (Supplementary Fig. 3d), the extrusion rate of the core solution is smaller than the sheath solution, where the winding speed could affect the sheath diameter. As a result, the sheath diameter could change according to the core diameter alteration. Notably, we found that the core fiber diameter almost matches the theoretical values based on the spinning parameter (Supplementary Fig. 3e), where the theoretical calculation of the core diameter is as follows:

$\text{Theoretical} D_{C} =2\times\sqrt{\frac{E_{C}}{S_{W}}}\times R_{C}$ (1)

where *D_C_* is the core diameter, *E_C_* represents the extrusion rate of the core solution, *R_C_* is the core diameter of the coaxial needle, and *S_W_* represents the winding speed.


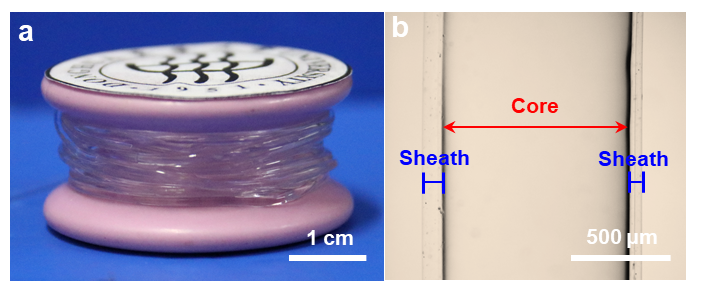


**Supplementary Figure 4.** (a) Photograph of a bobbin of NIPAm-*co*-DMAAm based core-sheath hydrogel fiber, (b) optical microscopy image of the NIPAm-*co*-DMAAm based core-sheath hydrogel fiber.


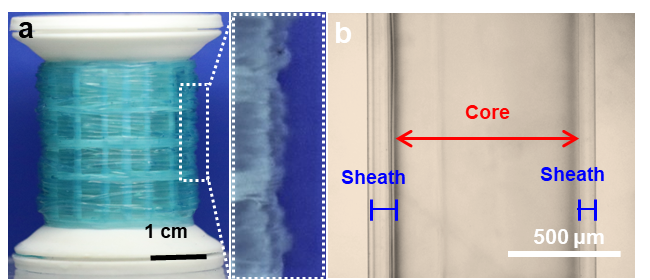


**Supplementary Figure 5.** (a) Photograph of a bobbin of (OEGMA-*co*-MEO_2_MA)/p(PEGDA-*co*-AAm)/Ca-alginate based semi-interpenetrating hydrogel fiber, (b) optical microscopy image of the (OEGMA-*co*-MEO_2_MA)/p(PEGDA-*co*-AAm)/Ca-alginate based semi-interpenetrating hydrogel fiber.

1. **The ^13^C NMR and FTIR Spectra of the Core Hydrogel Fiber**


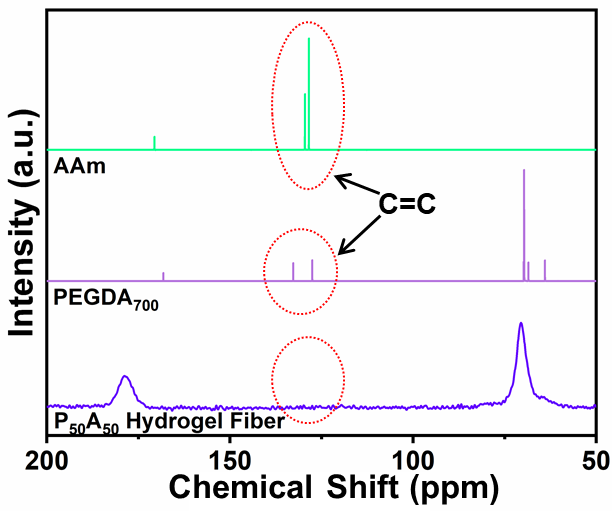


**Supplementary Figure 6.** ^13^C NMR spectra of AAm, PEGDA_700_ and P_50_A_50_ hydrogel fiber.


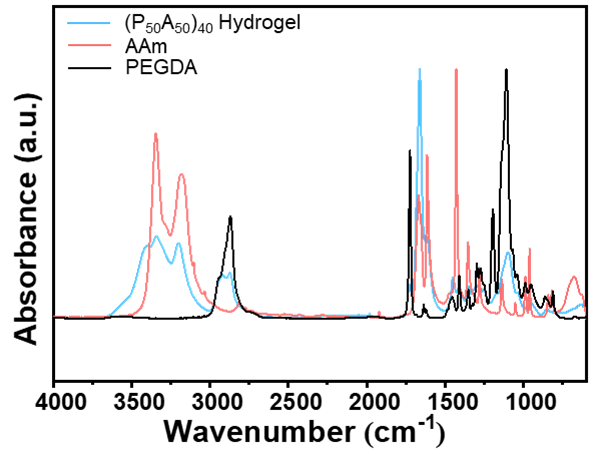


**Supplementary Figure 7.** The FTIR absorption spectra of PEGDA, AAm, and (P_50_A_50_)_40_ hydrogel.

1. **Fiber Microstructure Design for Optimal Waveguiding Properties**


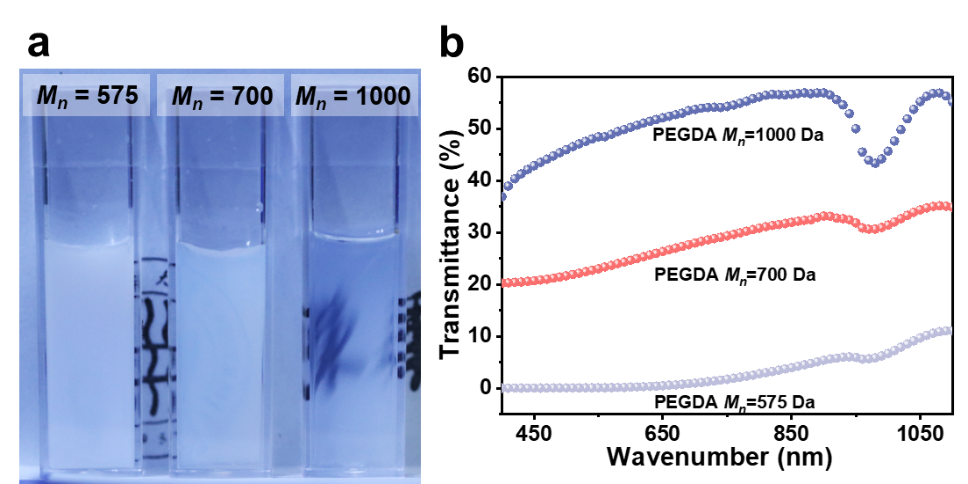


**Supplementary Figure 8.** (a) Photograph and (b) optical transmittance of PEGDA hydrogels with different molecular weight (*M_n_* = 575 Da, 700 Da, 1000 Da).


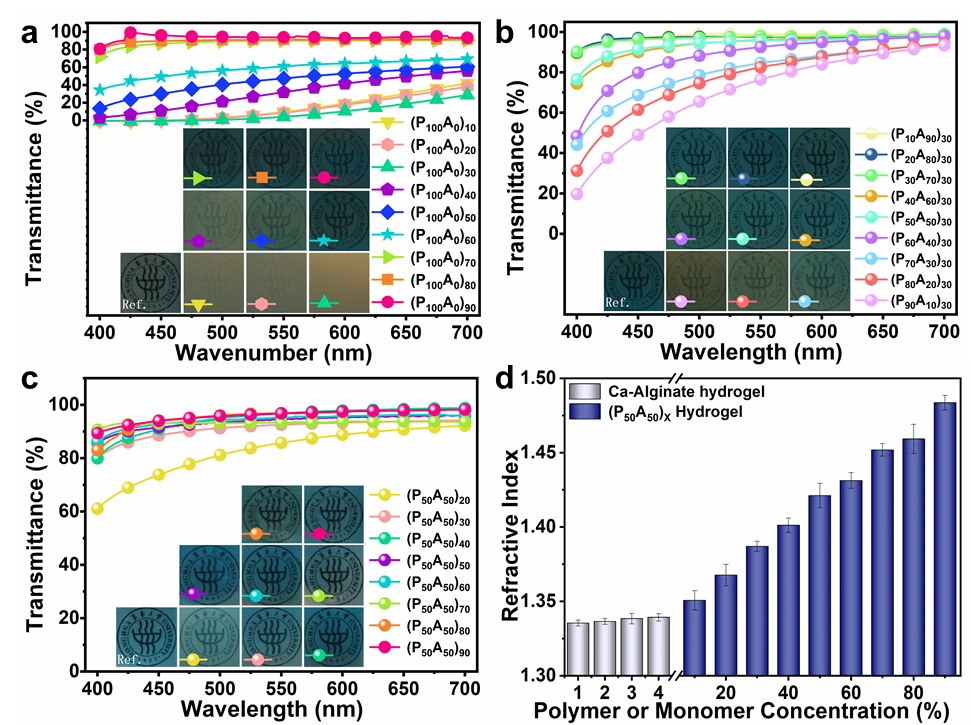


**Supplementary Figure 9.** Optical properties of selected materials: Transmission of (a) PEGDA at different concentration, (b) (P_a_A_100-a_)_30_ at different AAm content (monomer concentration was 30 wt%), (c) (P_50_A_50_)_X_ at different total monomer concentration; (d) Refractive index of (P_50_A_50_)_X_ hydrogels and Ca-alginate hydrogels at different monomer or polymer concentration.


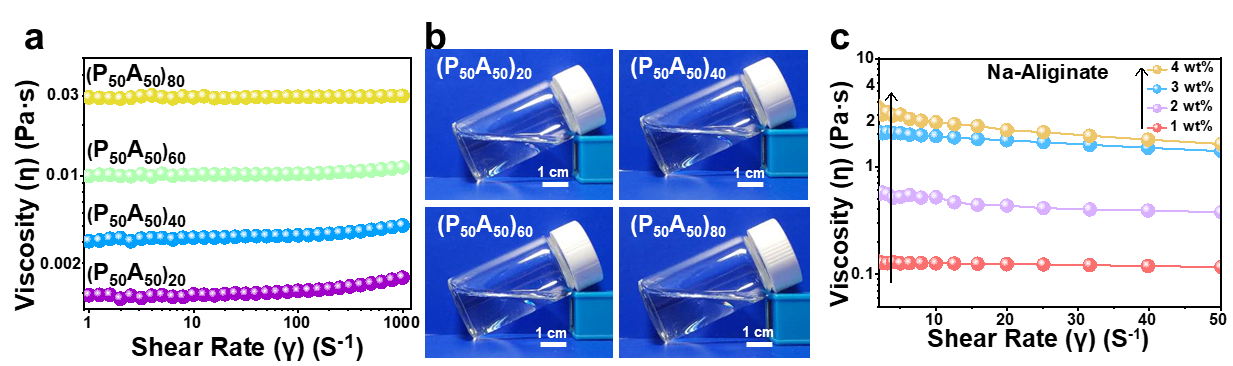


**Supplementary Figure 10.** (a) Viscosity and (b) photographs of (P_50_A_50_)_X_ solutions for different monomer concentrations, (c) viscosity of Na-alginate for different polymer concentration.

1. **The Evaluation of Light Propagation Property**


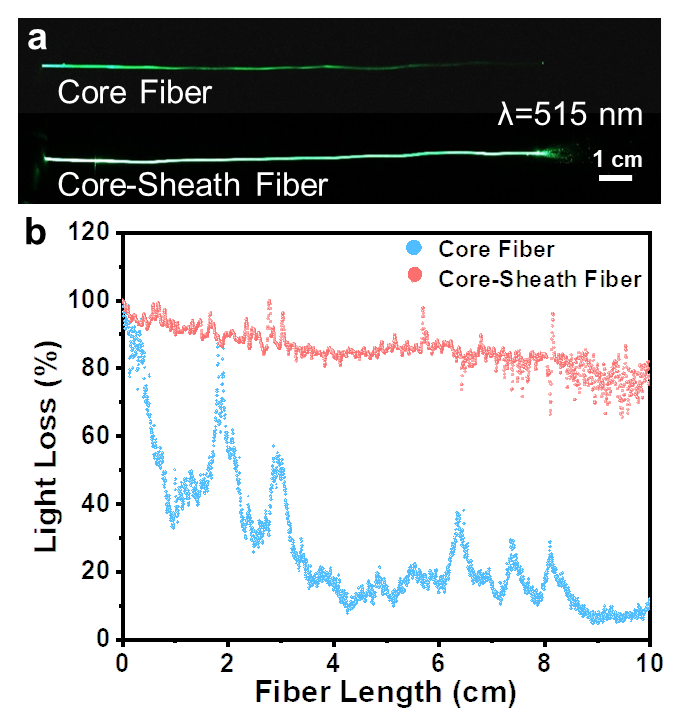


**Supplementary Figure 11.** (a) Photograph of light propagation through the a (P_50_A_50_)_40_-only core fiber and a (P_50_A_50_)_40_ core/Ca-alginate sheath fiber, (b) light loss through the core fiber and core-sheath fiber.

**
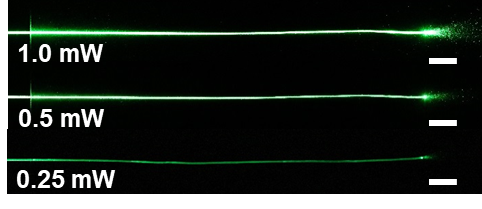
**

**Supplementary Figure 12.** Light transmission of a (P_50_A_50_)_40_ OWHF (541/845) with different laser intensities, the scale bar is 1 cm.

**
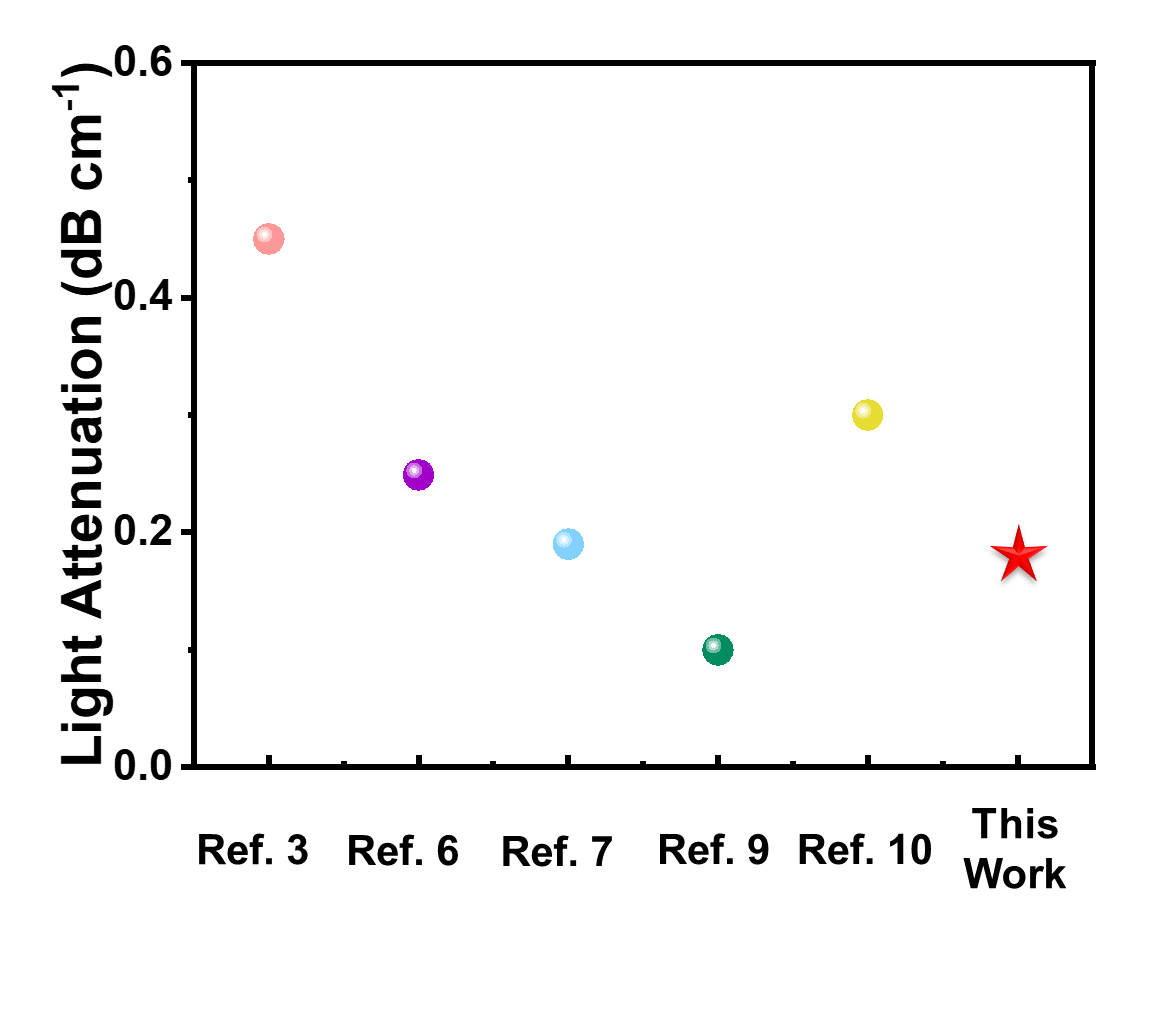
**

**Supplementary Figure 13.** Comparasion of light attenuation of OWHF in this work with other similar hydrogel optical fibers.





**Supplementary Figure 14.** Relative light intensity of OWHF under repeatable bending cycles for bending 180 ^o^.


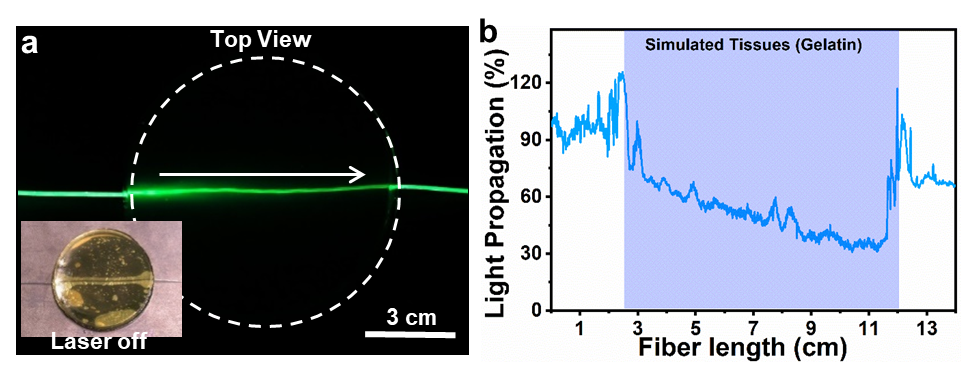


**Supplementary Figure 15.** (a, b) Light propagation of a (P_50_A_50_)_40_ OWHF (541/845) in the simulated tissue (gelatin).

^
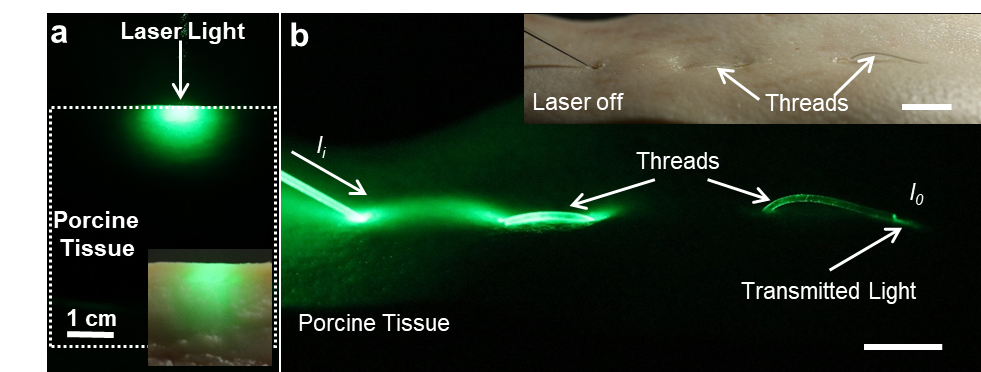
^

**Supplementary Figure 16.** (a) Light transmission in porcine tissue without implanted OWHF, the photo inset in (a) is taken under the environmental light was on; (b) A (P_50_A_50_)_40_ OWHF (541/845) implanted into porcine tissue *in vitro*, scale bar is 1 cm.


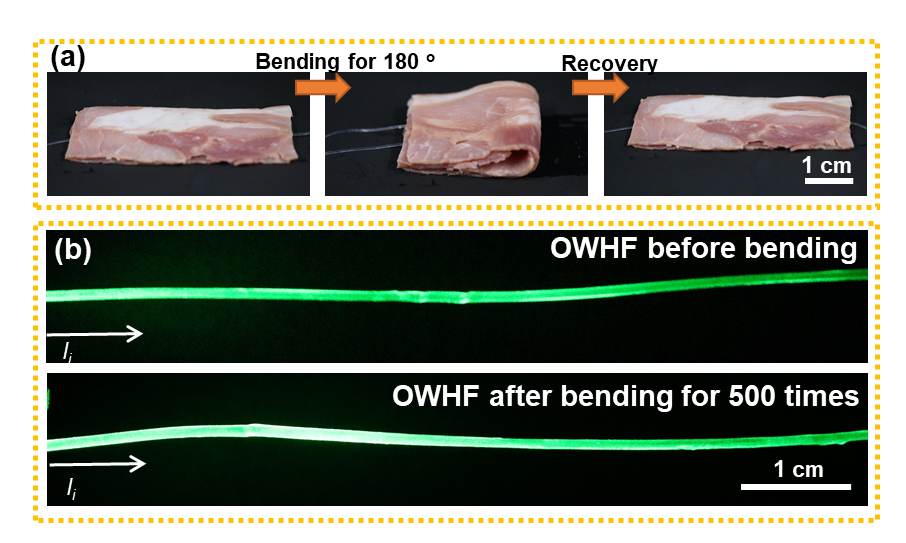


**Supplementary Figure 17.** (a) Photos of OWHF sandwich between two pieces of porcine tissues bending for 180^o^ and recovery; (b) Photos of light propagate through the OWHF before and after bending 180^o^ for 500 times, which OWHF was sandwich between two pieces of porcine tissues.

1. **The Mechanical Properties & Biocompatibility**





**Supplementary Figure 18.** Swelling behavior of (P_50_A_50_)_40_ OWHF as a function of times.

1. **Deep-tissue Photothermal Therapy**


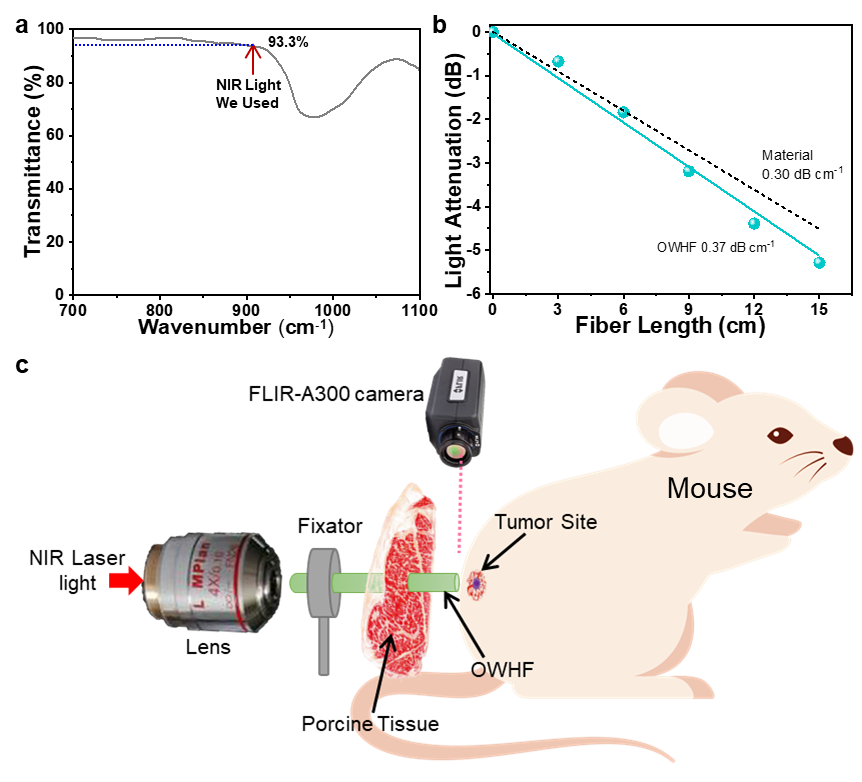


**Supplementary Figure 19.** (a) NIR light transmittance of (P_50_A_50_)_40_ hydrogel, (b) propagation NIR light loss in optical-waveguide hydrogel fiber compared to raw material, (c) simple illustration of homemade device for temperature change test of the *in vivo* photothermal cancer therapy under 915 nm NIR laser light through OWHF.

For the photothermal therapy of tumor deep in the tissue, the (P_50_A_50_)_40_ OWHF could be used for implantable light-guides to transport NIR light *in vivo*. First, we tested the transmittance at the near-infrared light through the (P_50_A_50_)_40_ hydrogel, and the result is shown in Supplementary Fig. 19a. It can be seen that there is about 93.5% of transmittance at 915 nm light through the hydrogel, and thus leading to a 0.37 dB cm^-1^ attenuation for the OWHF at this laser light (Supplementary Fig. 18b).

1. **Optogenetic Simulation for Brain-interface Communications**


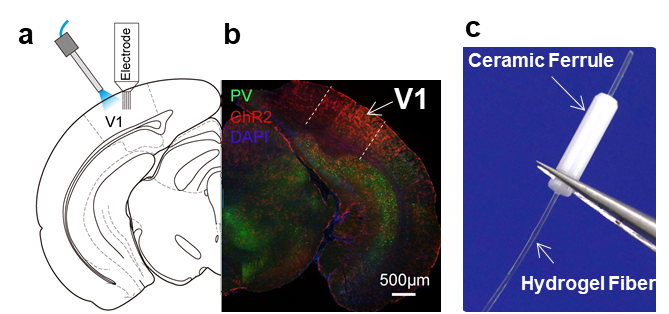


**Supplementary Figure 20.** Photographs of (a) a simple schematic illustration for recording of electrophysiological activities, and (b) expression of ChR2 (red) in the V1 of PV-ChR2 mice, (c) a (P_50_A_50_)_40_ OWHF coupled with ceramic ferrule.


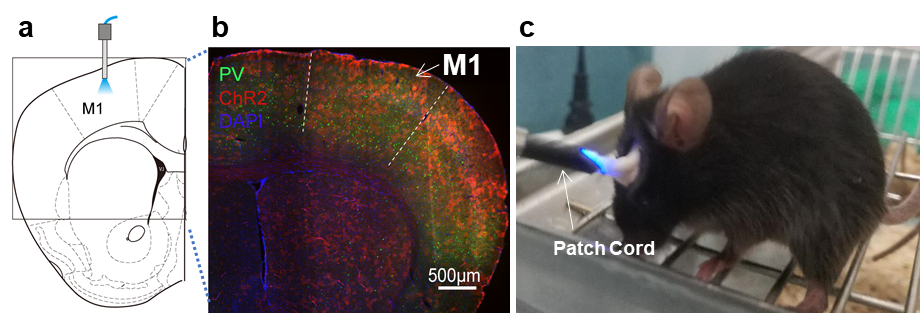


**Supplementary Figure 21.** (a) A simple schematic illustration for optogenetic stimulation *in vivo*, (b) expression of ChR2 (red) in the M1 of PV-ChR2 mice, and (c) photograph of a mouse under the optogenetic stimulation.


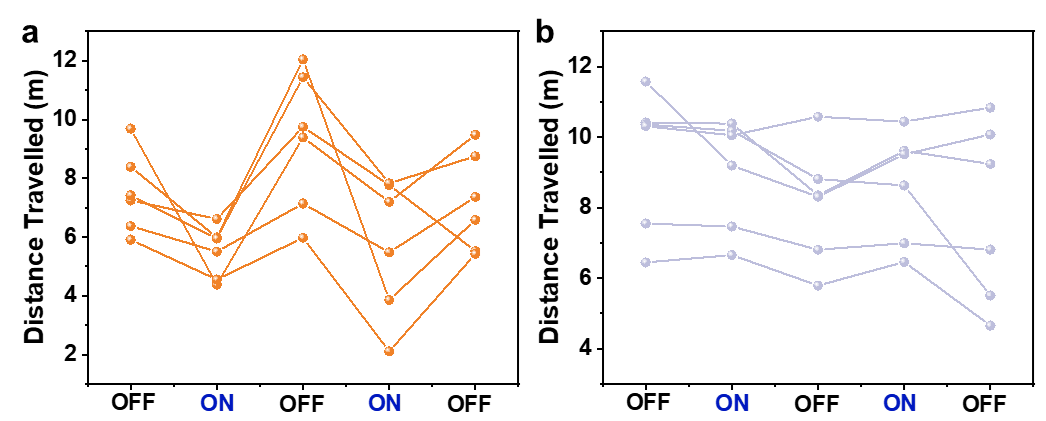


**Supplementary Figure 22.** Travelling distance of mice of (a) experimental and (b) controlled groups in the open field (total 15 min divided into 5 epochs, every epoch was 3 min).


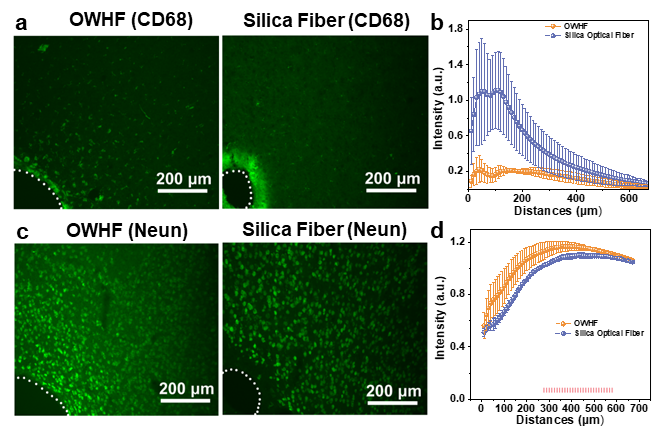


**Supplementary Figure 23.** Immune responses and neuron density in OWHF and silica fiber implanted cortical tissue four weeks after the implant surgery: (a-b) CD68 and (c-d) NeuN immunostaining of OWHF compared to conventional silica fiber after implanted for 4 weeks. Red bars in (b) and (d) indicated statistically significant data points.

**Supplementary References:**

S1. Choi M., *et al*. Step-index optical fiber made of biocompatible hydrogels. *Adv Mater* 2015; **27(27)**: 4081-6.

S2. Yetisen A. K., *et al*. Glucose-sensitive hydrogel optical fibers functionalized with phenylboronic acid. *Adv Mater* 2017; **29(15)**: 1606380.

S3. Jiang N., *et al*. Functionalized flexible soft polymer optical fibers for laser photomedicine. *Adv Opt Mater* 2018; **6(3)**: 1701118.

S4. Choi M., *et al.* Light-guiding hydrogels for cell-based sensing and optogenetic synthesis *in vivo*. *Nat Photonics* 2013; **7(12)**: 987-94.

S5. Zhong R., *et al*. Egg white-mediated green synthesis of CuS quantum dots as a biocompatible and efficient 980 nm laser-driven photothermal agent. *RSC Adv* 2016; **6(46)**: 40480-8.
